# Picosecond multi-hole transfer and microsecond charge-separated states at the perovskite nanocrystal/tetracene interface†
†Electronic supplementary information (ESI) available: Fig. S1–S9, sample preparations, TA experiment set-ups, and other supplementary contents. See DOI: 10.1039/c8sc04408b


**DOI:** 10.1039/c8sc04408b

**Published:** 2018-12-21

**Authors:** Xiao Luo, Guijie Liang, Junhui Wang, Xue Liu, Kaifeng Wu

**Affiliations:** a State Key Laboratory of Molecular Reaction Dynamics , Dynamics Research Center for Energy and Environmental Materials , Collaborative Innovation Center of Chemistry for Energy Materials (iChEM) , Dalian Institute of Chemical Physics , Chinese Academy of Sciences , Dalian , Liaoning 116023 , China . Email: kwu@dicp.ac.cn; b Hubei Key Laboratory of Low Dimensional Optoelectronic Materials and Devices , Hubei University of Arts and Science , Xiangyang , Hubei 441053 , China

## Abstract

Hole transfer (HT) is often kinetically sluggish compared to electron transfer (ET), which increases recombination losses and thus limits the efficiency of many energy conversion devices such as light-emitting diodes, solar cells and solar-fuel production systems. Recently introduced lead halide perovskites and their nanocrystals (NCs) have emerged as an important class of energy conversion materials. Here we report that tetracene molecules can enable ultrafast and efficient HT from perovskite NCs. Transient absorption measurements reveal that HT occurs in 7.6 ± 0.2 ps, and the charge-separated states are extremely long-lived (5.1 ± 0.3 μs). Such exceptional charge separation properties are leveraged to demonstrate the dissociation of up to 5.6 excitons per NC by multi-hole transfer for the first time. These results not only suggest that tetracenes are an effective hole-extracting material for perovskite devices, but also have important implications for using perovskite NCs as sensitizers and tetracenes as redox mediators to drive single and even multi-electron photochemical reactions.

## Abstract 2

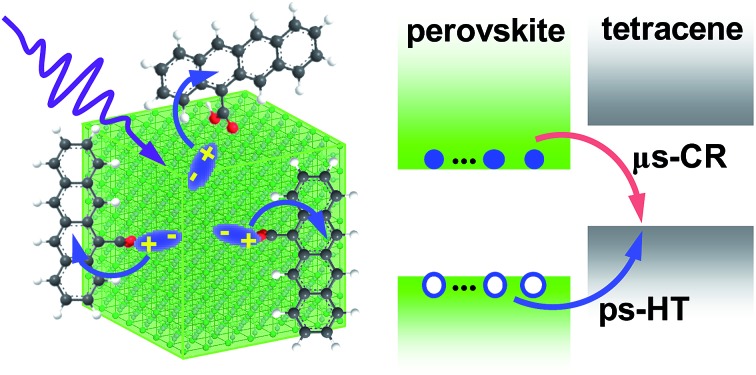
Picosecond hole transfer and microsecond charge-separated states were observed at the perovskite nanocrystal/tetracene interface, which are leveraged to demonstrate the dissociation of up to 5.6 excitons per nanocrystal by multi-hole transfer.

## Introduction

Lead halide perovskite nanocrystals (NCs) have emerged as a promising enabler for many optoelectronic applications.^1^,^2^ These NCs, upon synthesis, can attain exceptional light harvesting and emitting properties^3^–^5^ that have already been utilized to demonstrate high-efficiency light-emitting diodes^6^ and solar cells.^7^ Merging semiconductor NCs with functional organic molecules is an extensively explored strategy to achieve new optical and electronic functionalities.^8^–^10^ This strategy utilizes some fundamental kinetic processes such as charge and/or energy transfer between NCs and molecules. Charge transfer (CT) from NCs to molecular catalysts can be used to drive photochemical reactions for solar energy conversion; energy transfer (EnT), on the other hand, can funnel the excitation energy from a light-harvesting antenna into the reaction or light-emitting centers.

In particular, recently, there has been growing interest in Dexter-type triplet EnT in NC–acene hybrids due to the peculiar singlet fission (SF) and triplet–triplet annihilation (TTA) behaviors of acene molecules.^10^–^15^ Triplet EnT from photoexcited NCs to acenes followed by TTA on acenes can be deployed in photon upconversion;^13^,^16^,^17^ alternatively, molecular triplets on acenes generated by SF can be harvested by NCs for efficiency-doubled light conversion or emission.^11^,^12^ An under-explored EnT mechanism, however, is the Förster-type singlet EnT (FRET) from NCs to acenes, which capitalizes on the strong light-harvesting capability of NCs to sensitize the absorption of acenes. This can be followed by efficient SF on acenes to double the number of excitons (similar to the concept of “quantum cutting”) which can be harvested, for example, by other types of narrow-gap NCs for high-efficiency light conversion.

Our initial idea for this work was to observe FRET from perovskite NCs to tetracene molecules as the emission spectrum of CsPbBr_3_ NCs overlaps well with the absorption spectrum of tetracenes (∼500 nm). The enormously large extinction coefficients of CsPbBr_3_ NCs (on the order of 10^7^ cm^–1^ M^–1^ at 400 nm)^18^ along with their low trap density make them ideal light-harvesters and FRET donors. Spectroscopic measurements, however, showed no evidence of EnT, but rather pointed to ultrafast (7.6 ps) and near-unity hole transfer (HT) from perovskite NCs to surface-attached tetracenes. The resulting charge-separated states are exceptionally long-lived, with an average lifetime of 5.1 μs. This was leveraged to demonstrate, for the first time, multi-hole transfer from multiply excited NCs to acenes; up to 5.6 excitons can be dissociated from each NC.

## Result and discussion

**Fig. 1:**
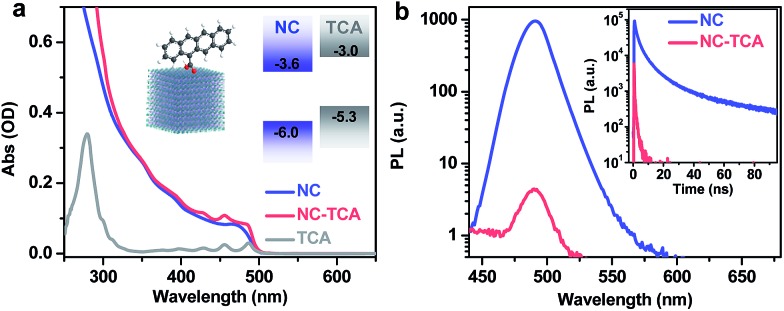
(a) Absorption spectra of perovskite NCs (blue solid line) and NC–TCA complexes (red solid line) dispersed in hexane. The difference between these two (gray solid line) is due to TCA absorption. The insets are the schematic structures and band alignments of NCs and TCAs. (b) PL spectra (main panel) and time-resolved PL decay curves (inset) of NCs (blue solid line) and NC–TCA complexes (red solid line) excited at 340 nm. Based on the absorption spectra in (a), NCs are selectively excited with 340 nm light.

CsPbCl_*x*_Br_3–*x*_ perovskite NCs with edge lengths within 8–10 nm (Fig. S1†) and tetracene carboxylic acid (TCA) molecules were synthesized according to the literature methods; see the ESI† for details. Cl was introduced to fine-tune the band gap of NCs so as to match their emission to the absorption of tetracenes (Cl to Br ratio ∼ 1 : 7; Fig. S2†). The carboxyl groups on TCAs should help anchor the tetracenes onto the NCs. NC–TCA complexes were prepared by a simple ligand-exchange procedure using sonication (see the ESI†). As the solubility of TCAs in hexane is negligible, the majority of TCAs remaining in the NC–TCA sample should be those bound to NC surfaces. Similar procedures have been extensively used in the literature to graft insoluble acceptor molecules onto colloidal quantum dots (QDs).^19^–^22^ Fig. 1a shows the absorption spectra of NCs and NC–TCA complexes dispersed in hexane. The difference between them is contributed by surface-attached TCAs. The extinction coefficient of NCs was measured to be 3.4 × 10^6^ M^–1^ cm^–1^ at 480 nm (see the ESI†), which is ∼3 orders-of-magnitude higher than that of TCAs (∼7.2 × 10^3^ M^–1^ cm^–1^), confirming the exceptional light-harvesting capability of perovskite NCs. Based on the absorption spectra and extinction coefficients, ∼137 TCAs are bound to each NC. The exact binding configuration has not been characterized in this work, but it is commonly assumed that similar polycyclic aromatic hydrocarbon (PAH) molecules bind to NC surfaces through the carboxyl groups.^14^,^15^,^23^

The schematic band alignment between NCs and tetracenes^11^ is shown in the inset of Fig. 1a. The values for NCs are simply those for bulk CsPbCl_*x*_Br_3–*x*_ with a Cl to Br ratio of ∼1 : 7 because of the very weak confinement of the NCs. Bulk CsPbCl_*x*_Br_3–*x*_ values are calculated as the composition-weighted average value using those reported for bulk CsPbCl_3_ (ref. 24) and CsPbBr_3_.^25^ Although the band edge positions of NCs are known to be sensitive to many factors such as surface ligands and are therefore difficult to accurately determine, because of the large energy offsets between NCs and TCAs (0.6–0.7 eV), the staggered alignment in the inset of Fig. 1a likely still holds. According to such an alignment, both EnT and HT from excited NCs to TCAs are energetically allowed. Photoluminescence (PL) measurements show that the emission of NCs is quenched by ∼300-fold in NC–TCA complexes (Fig. 1b). However, no TCA emission at ∼585 nm (Fig. S3†) is observed. Even if we account for the PL quantum yield (QY) difference between NCs and TCAs (25.0% *vs.* 2.15%), no sign of EnT can be detected. Note that the QY of TCAs was measured using 453 nm excitation light. Thus, HT completely dominates over EnT in NC–TCA complexes. PL lifetime measurements (Fig. 1b inset) indicate that the HT kinetics is barely detectable using our TCSPC set-up, suggesting that the HT time is ≪200 ps (instrument response function). In the following, we investigated the HT and charge recombination (CR) kinetics in detail using TA spectroscopy.

**Fig. 2:**
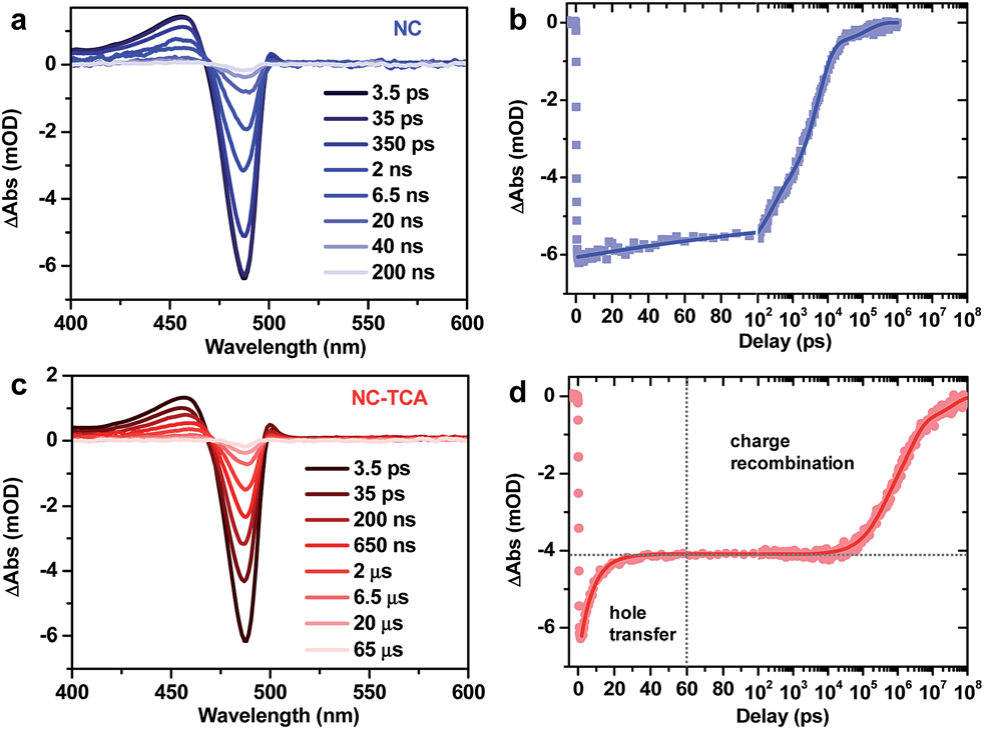
(a) TA spectra of NCs probed at indicated time delays following excitation with a 340 nm pulse (with (a) TA spectra of NCs probed at indicated time delays following excitation with a 340 nm pulse (with 〈*N*〉 ∼ 0.07). (b) TA kinetics of NCs probed at an exciton bleach (XB) center of ∼487 nm (blue squares) and its multi-exponential fit (blue solid line). (c and d) Similar plots to (a) and (b) for NC–TCA complexes. ∼ 0.07). (b) TA kinetics of NCs probed at an exciton bleach (XB) center of ∼487 nm (blue squares) and its multi-exponential fit (blue solid line). (c and d) Similar plots to (a) and (b) for NC–TCA complexes.

Details on TA set-ups can be found in the ESI.† The pump pulse was tuned to 340 nm to selectively excite the NCs, as the absorption spectra in Fig. 1a suggest that the absorption of NCs at this wavelength is ∼50-fold higher than that of TCAs. Fig. 2a shows the TA spectra of NCs excited with an average exciton number per NC ( shows the TA spectra of NCs excited with an average exciton number per NC (〈*N*〉) of ∼0.07, which are dominated by an exciton bleach (XB) feature due to the state-filling signals of band edge electrons and holes.) of ∼0.07, which are dominated by an exciton bleach (XB) feature due to the state-filling signals of band edge electrons and holes.^26^ The XB feature is long-lived (Fig. 2b), with 25% of its amplitude decaying with a time constant of 213 ± 15 ps, presumably due to carrier trapping, and 66% and 9% of its amplitude decaying with time constants of 5.3 ± 0.2 ns and 16.1 ± 1.8 ns, respectively, which can be assigned to radiative and/or nonradiative electron–hole recombination.

The TA spectra of the NC–TCA complexes are also dominated by the XB feature of NCs (Fig. 2c). However, their XB kinetics is clearly distinct from that of free NCs (Fig. 2d); ∼33% of its amplitude shows ultrafast decay within a few ps, whereas the other 67% is very long-lived, decaying on a μs timescale. The amplitudes of these two components (33% and 67%) are exactly the hole and electron contributions to the XB feature reported in a previous study;^26^ electron and hole contributions measured for our specific NC sample also give consistent results (Fig. S4†). On the basis of the PL and TA measurements, we can assign the ps component to HT from excited NCs to TCAs and the μs one to recombination of the charge-separated states (NC^–^–TCA^+^). Multi-exponential fit to the kinetics reveals that the HT process has a single-exponential time constant of 7.6 ± 0.2 ps and the CR process has an amplitude-averaged time constant of 5.1 ± 0.3 μs. Compared with the long XB lifetime in free NCs (Fig. 2b), the HT efficiency is virtually unity. The dominance of HT over EnT is likely a consequence of the very fast rate of HT. Indeed, the EnT time from NCs to TCAs estimated using the FRET model is 0.46 ns (ESI†), which is significantly slower than the measured HT time.

An alternative explanation for the observed fast XB decay is carrier trapping in some surface states introduced in the TCA ligand exchange process. However, it is very unlikely that the proportion of carriers being trapped (∼33%) coincides exactly with the hole contribution to the XB feature for these NCs. Even if this coincidence is true, the fact that the PL of NCs in the NC–TCA complexes is almost completely quenched (Fig. 1b) would require that the trapping process selectively and completely removed the holes from these NCs, which is even more unlikely to occur. As such, the most reasonable interpretation of the TA kinetics observed in the NC–TCA complexes is ultrafast HT from NCs to TCAs followed by slow recombination of charge separated states.

Note that HT kinetics can in principle also be analyzed using the ground-state-bleach of TCAs or absorption of TCA cations. However, because the extinction coefficient of TCAs is ∼3 orders-of-magnitude lower than that of NCs and because there is a spectral overlap between them, detection of TCA signals is technically difficult. TCA cations were reported to have broadband absorptions in the 500–900 nm range,^27^ but their extinction coefficients are also likely too weak to be detected compared to the NC signals. Indeed, a previous study on hole transfer from PbS QDs to TCA did not report the observation of TCA cations either.^15^ The situation is further complicated by the overlap between the TCA cation absorption and the broadband photoinduced absorption (PA) signals extensively reported for all types of NCs (QDs). As shown in Fig. S5,† in the low excitation regime, PA is ∼50-fold weaker than XB, which is possibly strong enough to surpass the TCA cation absorption feature. Nonetheless, measurements on several independently prepared NC samples give similar results (Fig. S6†), proving the robustness of the HT and CR kinetics reported here.

A possible reason for the ultrafast HT is the strong electronic coupling between NCs and TCAs. It was reported that Dexter EnT from pentacene triplets to PbSe NCs occurred in sub-ps^12^ and CT from PbS NCs to tetracene likely took only a few ps.^15^ As both CT and Dexter EnT rely on wavefunction exchanges between NCs and acenes, these results suggest that the electronic coupling between NCs and acenes is indeed generally strong. Our HT time compares favorably with previously reported HT times in various NC–molecule hybrids. For example, HT times from CsPbBr_3_ NCs to phenothiazine^26^ and 1-aminopyrene^28^ were reported to be ∼50 ps and ∼120 ps, respectively. Sub-ps HT from CdS NCs to PTZ functionalized with exciton-delocalizing ligands was reported,^29^ but only for a small proportion of NCs (<40%); the majority of holes are still transferred on a 10s to 100s of ps timescale. This raises another important novel aspect of HT to TCAs. Previous reports always showed highly heterogeneous HT kinetics, primarily due to the distribution of the number of molecular acceptors per NC.^8^,^30^,^31^ The single-exponential time constant observed for HT from NCs to TCAs suggests the absence of such a distribution, the reason for which remains unclear and warrants detailed investigations in the future. Our CR time of ∼5.1 μs is longer than most of the values reported for NC–molecule hybrids (1–100 ns)^19^,^32^–^34^ and is comparable to those achieved in nanorod systems which enable long-distance charge separation.^35^,^36^ The reason for this ultraslow CR is also unclear, but it probably implies that CR falls in the Marcus inverted region. Nonetheless, the homogeneous, ultrafast HT process, along with the extremely slow CR, suggests that tetracenes are an effective hole-extracting material for perovskite NCs.

We noticed that the HT time is much faster than the biexciton Auger recombination (AR) time (typically 10s of ps) reported for perovskite NCs,^26^,^37^–^44^ which encouraged us to test the possibility of multi-hole transfer from multiply excited NCs to TCAs. Multi-charge transfer is a challenging but effective way to drive multielectron photochemical reactions *via* bypassing the many intermediate recombination steps involved in sequential photon absorption triggered charge transfer reactions. It has been shown that interfacial electron transfer (ET) from NCs to attached molecules is fast enough to dissociate multiple excitons from NCs,^21^,^45^–^48^ but a similar process for HT has never been reported as HT is often kinetically sluggish compared to ET.

**Fig. 3:**
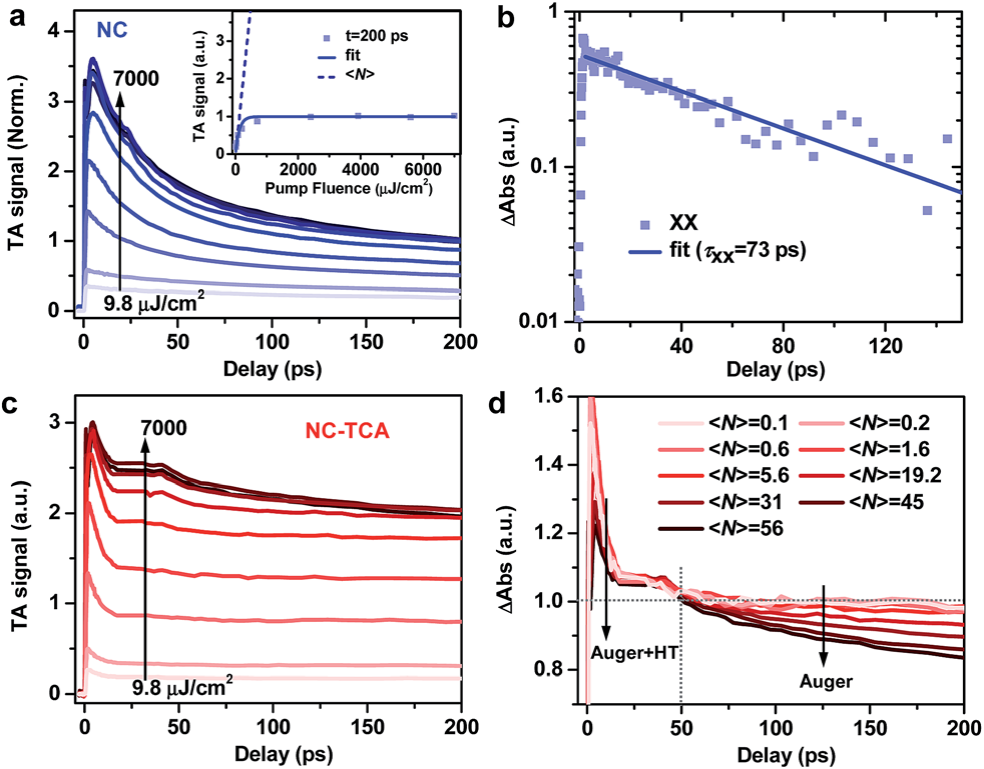
(a) Scaled TA kinetics probed at the XB of NCs excited with various 340 nm pump fluences. The inset shows the signal at a delay time of 200 ps as a function of excitation fluence (blue squares) and its fit to a Poisson model (blue solid line), and the average exciton numbers ((a) Scaled TA kinetics probed at the XB of NCs excited with various 340 nm pump fluences. The inset shows the signal at a delay time of 200 ps as a function of excitation fluence (blue squares) and its fit to a Poisson model (blue solid line), and the average exciton numbers (〈*N*〉; blue dashed line) at these fluences. (b) Biexciton recombination kinetics (blue squares) obtained by performing a subtraction between XB kinetics with 〈; blue dashed line) at these fluences. (b) Biexciton recombination kinetics (blue squares) obtained by performing a subtraction between XB kinetics with 〉; blue dashed line) at these fluences. (b) Biexciton recombination kinetics (blue squares) obtained by performing a subtraction between XB kinetics with 〈*N*〉 ∼ 0.6 and 〈 ∼ 0.6 and 〉 ∼ 0.6 and 〈*N*〉 ∼ 0.2 and its single-exponential fit (blue solid line). (c) Similar plot to (a) for NC–TCA complexes. The scaling factor for TA signals is the same as that for NCs in (a). (d) TA kinetics for NC–TCA complexes normalized in the range of 20–50 ps. ∼ 0.2 and its single-exponential fit (blue solid line). (c) Similar plot to (a) for NC–TCA complexes. The scaling factor for TA signals is the same as that for NCs in (a). (d) TA kinetics for NC–TCA complexes normalized in the range of 20–50 ps.

We measured multi-exciton dynamics in NCs using pump fluence-dependent TA (Fig. 3a); see Fig. S7† for TA spectra. By fitting the signal amplitudes at 200 ps, when the fast AR component has finished, to a Poisson model (Fig. 3a inset),^49^ we estimate the average exciton number we estimate the average exciton number 〈*N*〉 at each pump fluence (see the ESI at each pump fluence (see the ESI†). Biexciton recombination kinetics is obtained by performing a subtraction between kinetic traces with ). Biexciton recombination kinetics is obtained by performing a subtraction between kinetic traces with 〈*N*〉 ∼ 0.6 and 〈 ∼ 0.6 and 〉 ∼ 0.6 and 〈*N*〉 ∼ 0.2 ( ∼ 0.2 (Fig. 3b), which can be fitted to a single-exponential time constant (*τ*_XX_) of 73.2 ± 3.4 ps. Using a well-established statistical scaling law for AR, the lifetime of an *N*-exciton state is obtained as follows: *τ*_NX_ = 2*τ*_XX_/*N*(*N* – 1).^50^–^52^ Thus, the HT time from NCs to TCAs is comparable to 5-exciton AR time (∼7.3 ps). Assuming a constant HT time and sequential, staircase-like multi-exciton AR, we estimate that at most ∼6.5 excitons per NC can be dissociated by HT (see the ESI and Fig. S8†). This is valid for the case of simultaneous multi-hole transfer. In the case of sequential multi-hole transfer, however, HT rate should be retarded by previous dissociated excitons as the electrons accumulated in NCs result in additional charging energy barrier for HT and HT has to compete with Auger recombination of charged multiexcitons. This coulombic energy effect on charge transfer was previously reported for C_60_ buckyballs^53^,^54^ and for QDs.^55^,^56^ Therefore, the simple calculation above sets an upper limit for the number of excitons that can be dissociated by HT.

Fig. 3c shows the pump fluence-dependent XB kinetics in the NC–TCA complexes measured under exactly the same conditions and scaled using the factor as those in Fig. 3a; see Fig. S9† for TA spectra. Ideally, the number of dissociated excitons can be analyzed using the signal amplitudes of the products (*i.e.*, TCA cations), as has been done in previous multi-electron transfer studies.^21^,^45^–^47^ However, the aforementioned PA signal of NCs overlapping with TCA cation absorptions also strongly increases with excitation fluences (Fig. S9†), prohibiting the extraction of TCA cation signal amplitudes.

The distinguishing kinetics features of HT and AR on different timescales offer an alternative way to determine the number of dissociated excitons. As shown in Fig. 3d, distinct from the progressively increasing AR signal observed for free NCs, the AR signal is strongly suppressed by HT kinetics. In Fig. 3d, we normalize the kinetic traces at different , we normalize the kinetic traces at different 〈*N*〉 in the range of 20–50 ps. In the <20 ps range, increasing 〈 in the range of 20–50 ps. In the <20 ps range, increasing 〉 in the range of 20–50 ps. In the <20 ps range, increasing 〈*N*〉 leads to faster decay due to the superposition of AR with HT; note that the smaller initial signal amplitude observed for very large 〈 leads to faster decay due to the superposition of AR with HT; note that the smaller initial signal amplitude observed for very large 〉 leads to faster decay due to the superposition of AR with HT; note that the smaller initial signal amplitude observed for very large 〈*N*〉 is a result of fast decay convoluted with slow XB formation at very high fluences possibly due to the hot-phonon-bottleneck reported for perovskites and their NCs. is a result of fast decay convoluted with slow XB formation at very high fluences possibly due to the hot-phonon-bottleneck reported for perovskites and their NCs.^57^–^59^ In the >50 ps range, the kinetics remains essentially the same for In the >50 ps range, the kinetics remains essentially the same for 〈*N*〉 from 0.1 to 5.6, beyond which the AR kinetics becomes progressively prominent. This directly evidences that HT can dissociate up to 5.6 excitons per NC: when 〈 from 0.1 to 5.6, beyond which the AR kinetics becomes progressively prominent. This directly evidences that HT can dissociate up to 5.6 excitons per NC: when 〉 from 0.1 to 5.6, beyond which the AR kinetics becomes progressively prominent. This directly evidences that HT can dissociate up to 5.6 excitons per NC: when 〈*N*〉 is below the maximum dissociation number, all the excitons are dissociated by HT and no AR feature can be observed; in contrast, when 〈 is below the maximum dissociation number, all the excitons are dissociated by HT and no AR feature can be observed; in contrast, when 〉 is below the maximum dissociation number, all the excitons are dissociated by HT and no AR feature can be observed; in contrast, when 〈*N*〉 exceeds the maximum dissociation number, the remaining, undissociated excitons decay exceeds the maximum dissociation number, the remaining, undissociated excitons decay *via* AR whose kinetics features in the 50–200 ps range can be detected.

This measured number (5.6) is lower than the estimated one (6.5), most likely because the HT rate decreases when the dissociated exciton number increases, as discussed above. However, a small deviation between them suggests that the charging energy effect for the NCs here is not as strong as those reported for C_60_ and QDs. This difference likely arises from the size effect on coulombic charging energy. The NCs used here have a diameter of ∼10 nm; the charging energy of introducing electrons into these NCs dispersed in a nonpolar solvent such as hexane was estimated to be ∼75 meV according to a previous report.^44^ The driving force for HT from NCs to TCAs, although difficult to accurately determine, is ∼0.7 eV (Fig. 1a inset). According to previous driving-force-dependent studies on charge transfer from NCs,^60^–^62^ for this driving force, a charge of 75 meV barely affects charge transfer rates. As a result, the HT rate stays relatively constant during the course of multi-HT from perovskite NCs.

## Conclusions

In summary, we observed 7.6 ps HT from perovskite NCs to surface-attached tetracenes and exceptionally long-lived (5.1 μs) charge-separated states. This ultrafast HT was used to demonstrate dissociation of up to 5.6 excitons from each NC. These results demonstrate that small acene molecules are very effective hole scavenging materials for perovskite NCs. This system may find applications in redox-mediated photo-oxidation for organic synthesis because acenes have been demonstrated to be hole transfer mediators *via* their reversible one-electron oxidation to radical cations.^63^,^64^ The multi-hole transfer dynamics also provides important implications for using perovskite NCs to drive multi-electron photochemical reactions.

## Conflicts of interest

The authors declare no conflict of interest.

## Supplementary Material

Supplementary information
