# Supplementary material for: Picosecond multi-hole transfer and microsecond charge-separated states at the perovskite nanocrystal/tetracene interface
Source: Chem Sci. 2018 Dec 21;10(8):2459–64. doi: 10.1039/c8sc04408b (PMC6385846; doi:10.1039/c8sc04408b)
Supplement: Supplementary file 1 [file SC-010-C8SC04408B-s001.pdf]

Electronic Supplementary Material (ESI) for Chemical Science.
This journal is © The Royal Society of Chemistry 2019

Electronic Supplementary Information for:

Picosecond Multi-Hole Transfer and Microsecond Charge-Separated States at the Perovskite Nanocrystal/Tetracene Interface

Xiao Luo, [a,†] *Guijie Liang,* [ab,†] *Junhui Wang,*[a] *Xue Liu,*[a] *and Kaifeng Wu*[a*]

[a] *State Key Laboratory of Molecular Reaction Dynamics, Dynamics Research Center for Energy and Environmental Materials, and Collaborative Innovation Center of Chemistry for Energy Materials (iChEM), Dalian Institute of Chemical Physics, Chinese Academy of Sciences, Dalian, Liaoning 116023, China*

[b] *Hubei Key Laboratory of Low Dimensional Optoelectronic Materials and Devices, Hubei University of Arts and Science, Xiangyang, Hubei 441053, China*

* Corresponding Author: kwu@dicp.ac.cn

† Xiao Luo and Guijie Liang contributed equally to this work.

Content list:

Figures S1-S9

Sample preparations

TA experiment set-ups

Estimation of the number of attached TCA molecules per NC

Estimation of FRET rate

Estimation of average exciton number at each excitation density

Estimation of exciton dissociation number

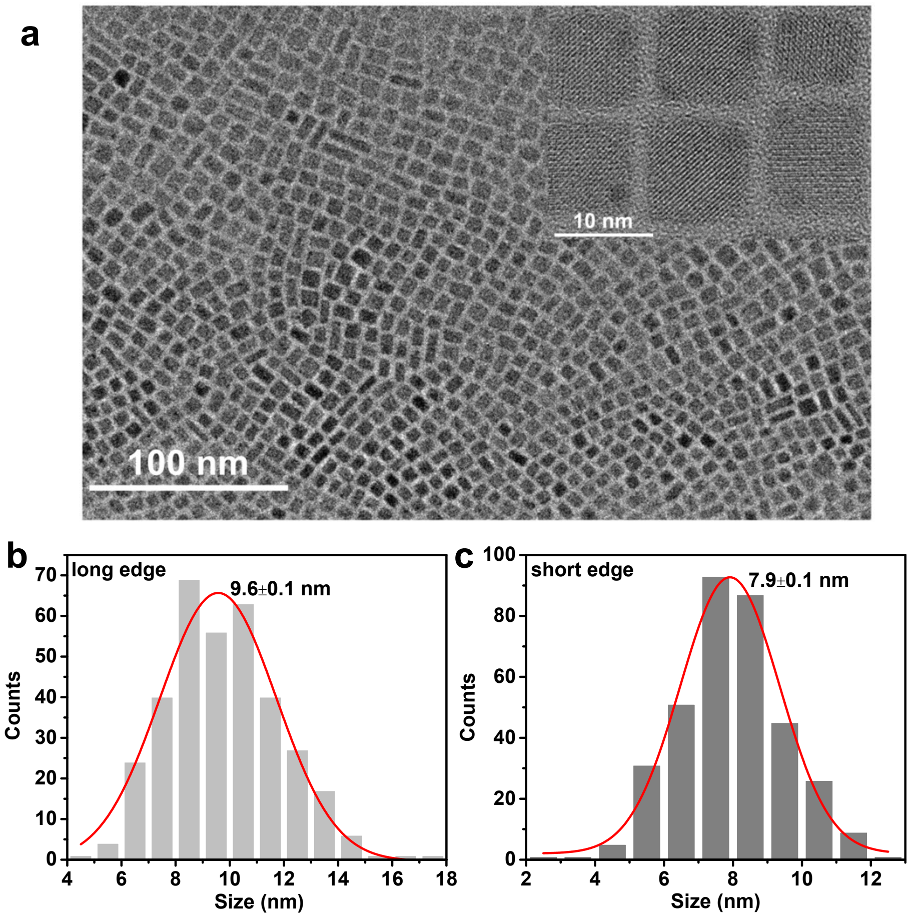

Figure S1. (a) A representative transmission electron microscopy (TEM) image of $CsPbBr_xCl_{3-x}$ perovskite NCs used in this study. Inset: high-resolution TEM. (b,c) Length distribution histograms of the NCs along the long (b) and short (c) edges.

a

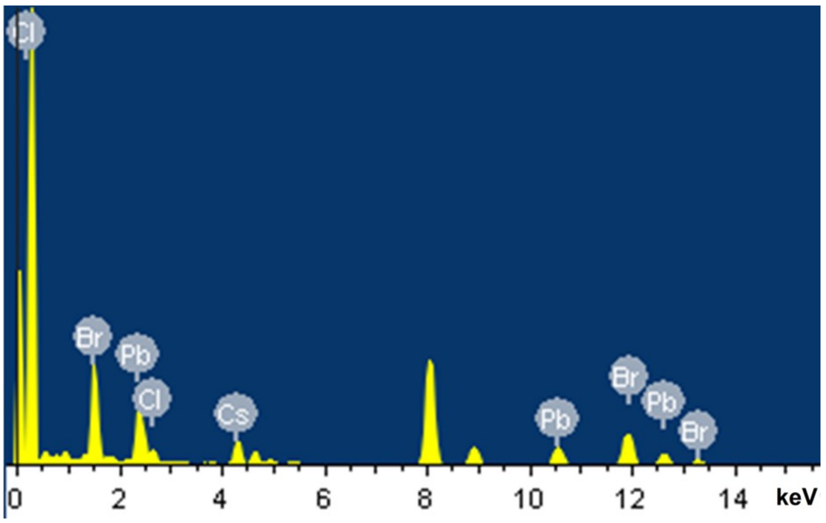

b

Element	Weight%	Atomic%
Cl K	2.46	7.75
Br L	37.77	52.87
Cs L	23.52	19.80
Pb M	36.25	19.57
Totals	100.00	

Figure S2. (a) Energy-dispersive x-ray (EDX) spectra of $CsPbBr_xCl_{3-x}$ Perovskite NCs. (b) Statistics of atomic ratios in NCs.

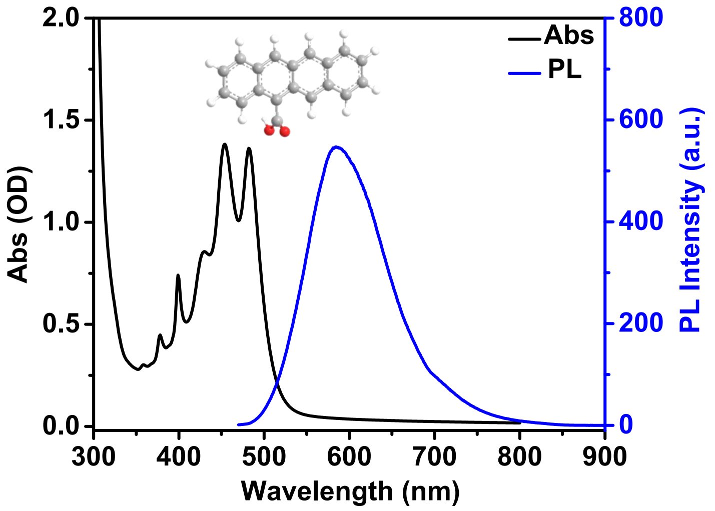

Figure S3. Absorption (black) and PL (blue) spectra of TCAs dissolved in toluene.

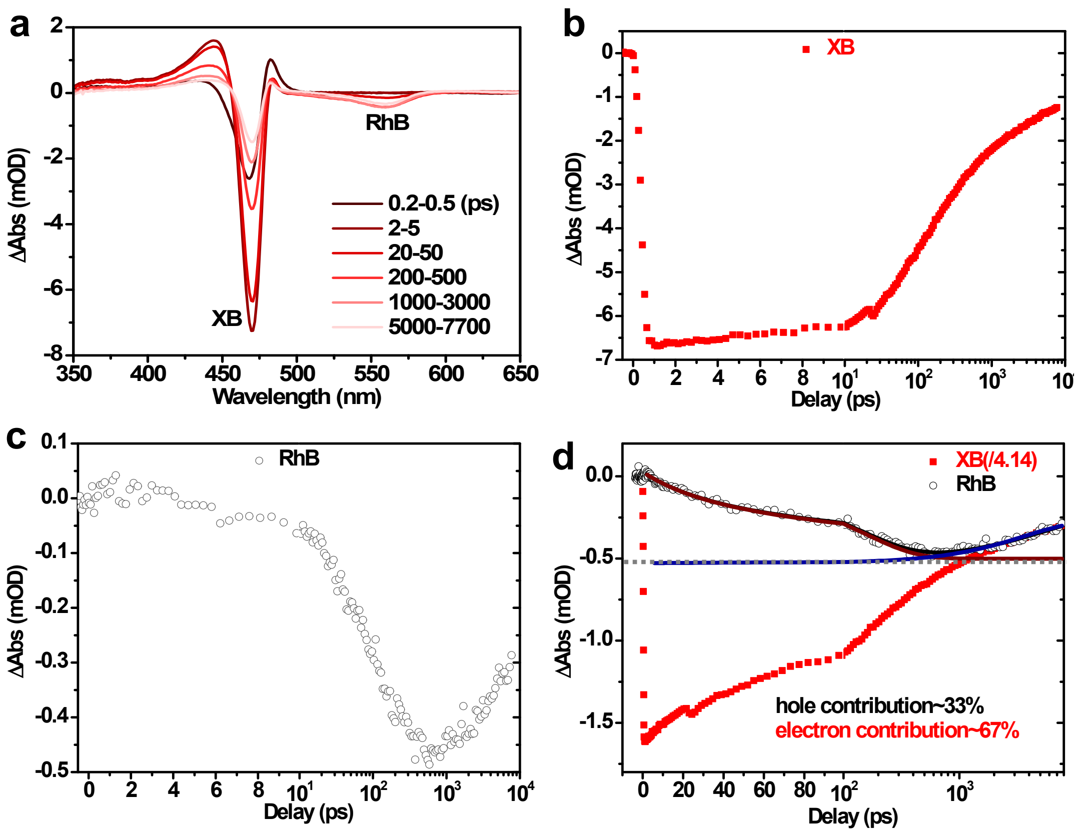

Figure S4. (a) TA spectra of NC-Rhodamine B complexes probed at indicated delays following the excitation by a 400 nm pulse which selectively excites NCs. (b,c) TA kinetics probed at the XB (b) and Rhodamine B ground-state-bleach (GSB) features. (d) Comparison of XB and RhB kinetics after scaling them to the same slowly-decaying tail. The formation of the RhB signal is complementary to the decay of the XB signal of NCs within 1000 ps; after that, they show the same slow decay on the ns timescale. According to previous reports,[1, 2] the fast and slow components can be assigned to electron transfer from photoexcited NCs to RhBs and charge recombination between RhB^- and NC^+, respectively. On the basis of this assignment, the electron and hole contributions to the XB of NCs can be determined. Specifically, fitting the RhB kinetics to multi-exponential function (black solid line) reveals its formation (wine solid line) and decay (blue solid line) components which are slightly convoluted with each other. The full amplitude of the formation and decay components

(~0.53 mOD) is the hole contribution to the XB of NCs (~1.6 mOD), which is ~33%. The remaining 67% is then contributed by electrons.

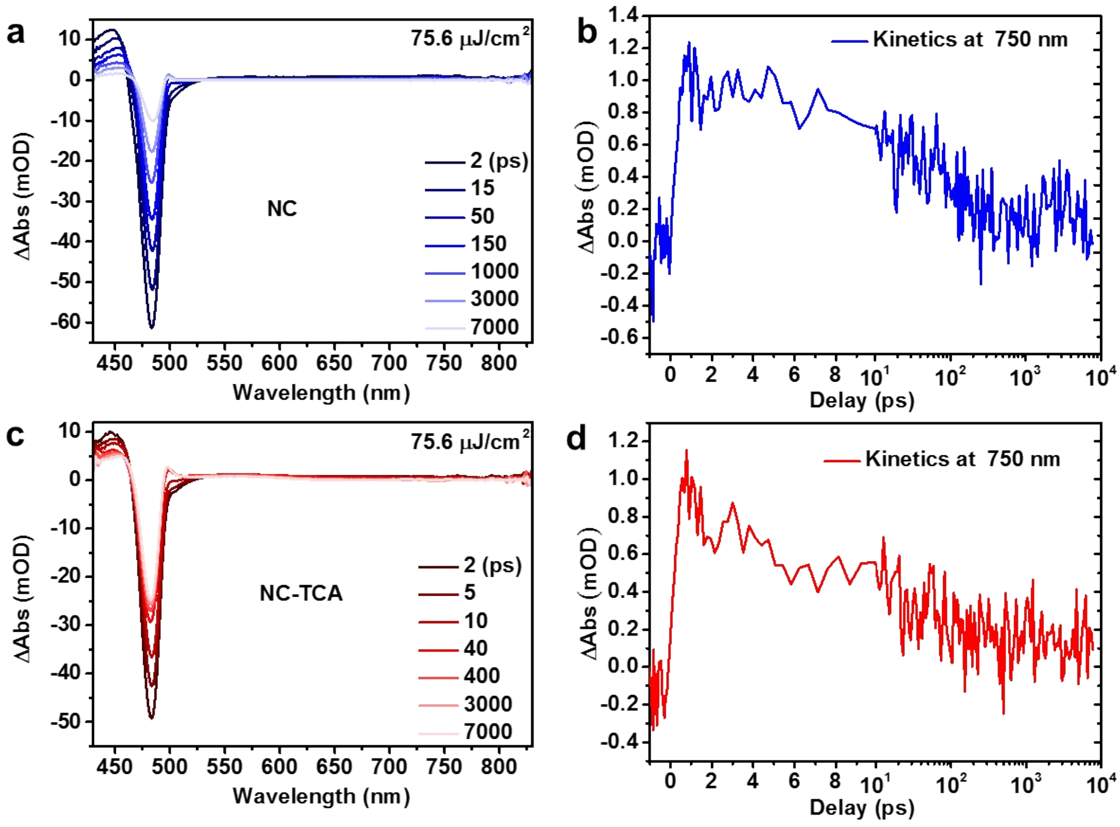

Figure S5. (a) TA spectra of NCs at varying delays following the excitation by a 340 nm with fluence of 75.6 μJ/cm^2. (b) TA kinetics probed at ~750 nm. (c,d) The same plots as (a) and (b) for NC-TCA complexes.

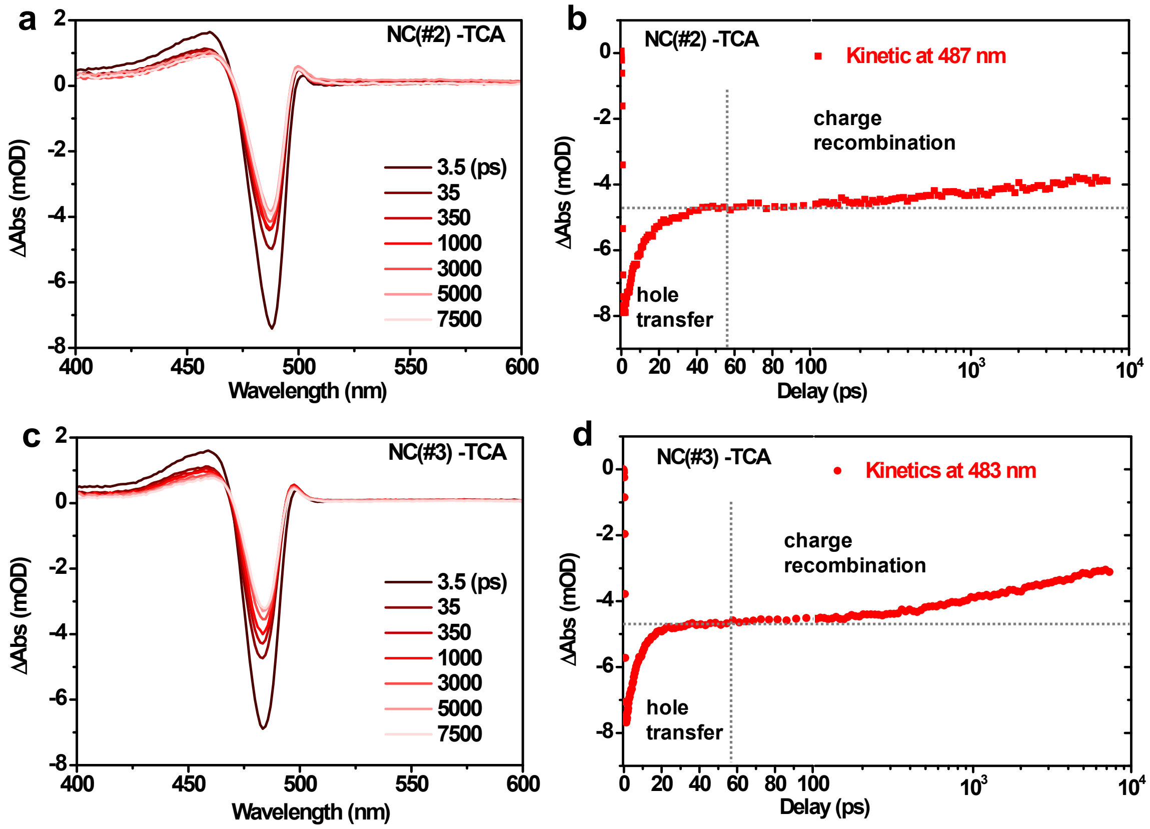

Figure S6. (a) TA spectra of NC(#2)-TCA complexes probed at indicated time delays following the excitation by a 340 nm pulse which selectively excites NCs. (b) TA kinetics probed at the XB center of NCs (~487 nm). (c,d) Similar plots as (a) and (b) but for NC(#3)-TCA complexes.

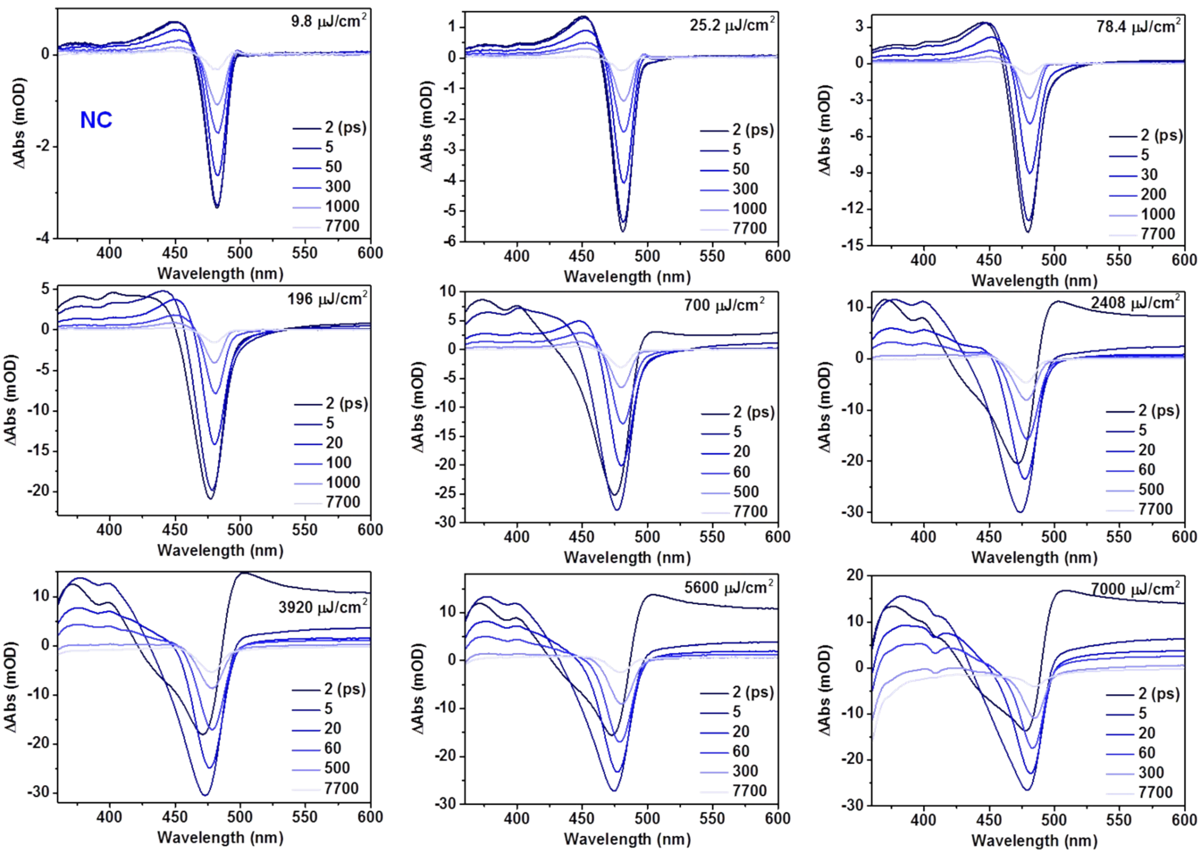

Figure S7. TA spectra of NCs probed at indicated time delays following the excitation by 340 nm pulses with various excitation densities from 9.8 to 7000 μJ/cm^2. Note that in analyzing the XB kinetics, we have subtracted the contribution of the photoinduced absorption (PA) signal by assuming that it has similar signal amplitudes across the probed wavelength range.

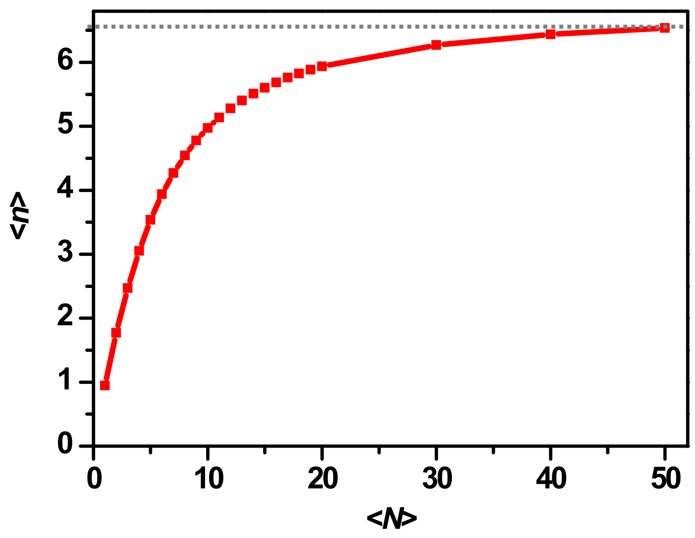

Figure S8. Estimated average number of dissociated excitons <*n*> as a function of average exciton number <*N*>. It saturates at a value of ~6.5.

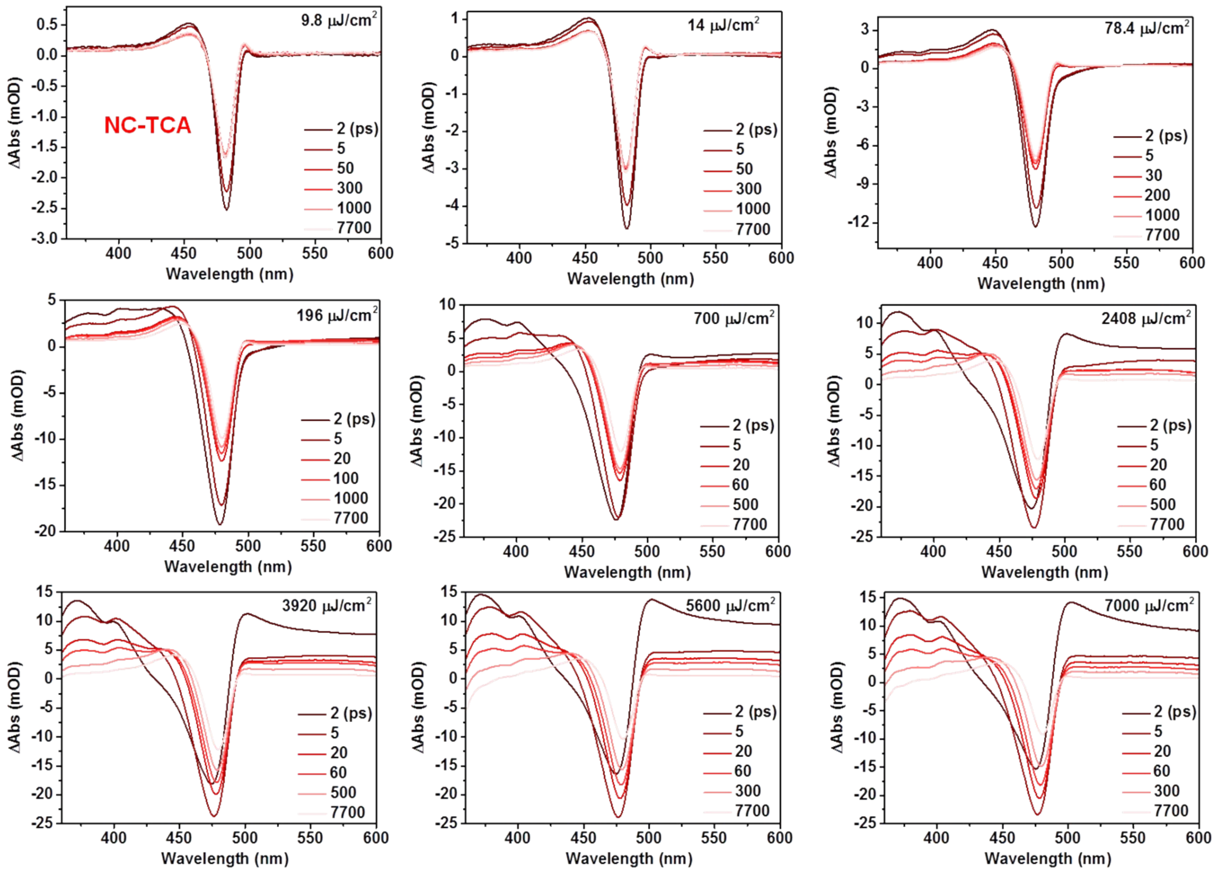

Figure S9. TA spectra of NC-TCA complexes probed at indicated time delays following the excitation by 340 nm pulses with various excitation densities from 9.8 to 7000 μJ/cm^2. Note

that in analyzing the XB kinetics, we have subtracted the contribution of the photoinduced absorption (PA) signal by assuming that it has similar signal amplitudes across the probed wavelength range.

Sample preparations

Synthesis of $CsPbBr_xCl_{3-x}$ NCs. $CsPbBr_xCl_{3-x}$ perovskite NCs were synthesized by using a hot injection approach with modifications.[3] In a typical reaction, 0.407 g Cs_2CO_3, 1.5 mL oleic acid (OA), and 20 mL octadecene (ODE) were degassed for 30 min under vacuum at 60 ºC, and heated under Ar atmosphere to 150 ºC until all the Cs_2CO_3 powders were dissolved. This CsOA precursor solution was kept at 100 ºC for use. A mixture of 0.202 g $PbBr_2$, 0.056 g $PbCl_2$ and 20 mL ODE were degassed at 120 ºC for 60 min, which was then switched to Ar atmosphere. 2.0 mL dried oleylamine (OLAM) and 2.0 mL dried OA were injected in to the mixture. The reaction temperature was raised to 180 ºC after a clear solution was obtained. At this temperature, 1.6 mL CsOA solution was swiftly injected. After 5 seconds, the reaction mixture was cooled down to room temperature by an ice-water bath. The NCs were separated from the crude solution by centrifuging at 7830 rpm for 10 mins. The precipitant was re-dispersed in 5 mL hexane by shaking and sonication, followed by centrifugation at 7830 rpm for 3 min in order to remove larger NCs and agglomerates.

Synthesis of 5-Tetracene carboxylic Acid (TCA). Synthesis of 5-Bromotetracene:[4] a solution of N-bromosuccinimide (354 mg, 2 mmol) in dry DMF (40 mL) was added during 1 h into a stirred at 60 °C solution of tetracene (456 mg, 2 mmol) in chloroform (200 mL). The

resulting solution was stirred at 60 °C for 3 h. After cooling to room temperature, the reaction mixture was washed with water (2×200 mL) and then chloroform was evaporated in Vacuo. The residue was diluted with water (200 mL), and the precipitate was filtered and dried, giving an orange powder (5-Bromotetracene). For analytical purposes, it was purified by column chromatography on silica gel using dichloromethane as eluent. Synthesis of TCA:[5] a solution of 5-bromotetracene (4.00 g, 13.0 mmol) in THF (130 mL) was added a n-hexane solution of nBuLi (1.60 M, 8.9 mL, 14.2 mmol, 1.1 equiv) dropwise at -78 ºC. After stirring for 1 h, some pellets of dry ice were added under a stream of argon. The mixture was kept at the same temperature for additional 30 min and then was allowed to warm to room temperature. A small amount of distilled water was added and solvents were concentrated under reduced pressure to obtain yellow solids, which were dissolved in aqueous sodium hydroxide solution (1.0 M, ca. 700 mL). The insoluble solids were then filtered off. The filtrate solution was acidified with aqueous hydrogen chloride solution (2.0 M) to precipitate orange solids, which were dried over phosphorus (V) oxide under reduced pressure to give 2.96 g (10.9 mmol) of TCA in 84% yield as an orange powder.

Preparation of NC-TCA complexes. The NC-TCA complexes were prepared by adding TCA powders into a NC solution in hexane, followed by sonication for 5 mins. The mixture was filtered to obtain a clear solution containing NC-TCA complexes; because the solubility of TCA in hexane is negligible, all the TCA molecules were believed to be bound to NC surfaces.

TA experiment set-ups

Femtosecond TA. The femtosecond pump-probe TA measurements were performed using a regenerative amplified Ti:sapphire laser system (Coherent; 800 nm, 70 fs, 6 mJ/pulse, and 1 kHz repetition rate) as the laser source and a femto-TA100 spectrometer (Time-Tech Spectra). Briefly, the 800 nm output pulse from the regenerative amplifier was split in two parts with a 50% beam splitter. The transmitted part was used to pump a TOPAS Optical Parametric Amplifier (OPA) which generated a wavelength-tunable laser pulse from 250 nm to 2.5 μm as pump beam. The reflected 800 nm beam was split again into two parts. One part with less than 10% was attenuated with a neutral density filter and focused into a 2 mm thick sapphire window to generate a white light continuum (WLC) used for probe beam. The probe beam was focused with an Al parabolic reflector onto the sample. After the sample, the probe beam was collimated and then focused into a fiber-coupled spectrometer with CMOS sensors and detected at a frequency of 1 KHz. The intensity of the pump pulse used in the experiment was controlled by a variable neutral-density filter wheel. The delay between the pump and probe pulses was controlled by a motorized delay stage. The pump pulses were chopped by a synchronized chopper at 500 Hz and the absorbance change was calculated with two adjacent probe pulses (pump-blocked and pump-unblocked). The samples were placed in 1 mm airtight cuvettes in a N_2-filled glove box and measured under ambient conditions. Samples were vigorously stirred in all the measurements.

Nanosecond TA. Nanosecond TA was performed with the EOS spectrometer (Ultrafast Systems LLC). The pump beam at 340 nm is generated in the same way as the femtosecond TA experiment described above. A different white light continuum (380-1700 nm, 0.5 ns pulse width, 20 kHz repetition rate) was used, which was generated by focusing a Nd:YAG

laser into a photonic crystal fiber. The delay time between the pump and probe beam was controlled by a digital delay generator (CNT-90, Pendulum Instruments). The probe and reference beams were detected with multichannel spectrometers and the same procedure was followed to measure the TA spectra and kinetics.

Estimation of the number of attached TCA molecules per NC

The number of attached TCA molecules per NC was estimated to be ~137 based on the measured absorption spectra and molar extinction coefficient of $CsPbBr_xCl_{3-x}$ NCs (3.4×10^6 $M^{-1}cm^{-1}$ at 480 nm) and TCA (7.2×10^3 $M^{-1}cm^{-1}$ at 480 nm). The molar extinction coefficient of TCA was measured from its dilute solution in toluene, which was used to calculate the molecular concentration of TCA in the complexes using Lambert-Beer law. The extinction coefficient of $CsPbBr_xCl_{3-x}$ NC was determined as follow. Firstly, the number of lead atoms per NC (n_1) is calculated based on the NC and unit-cell volumes of $CsPbBr_xCl_{3-x}$. In addition, total lead atoms concentration (n_2) of $CsPbBr_xCl_{3-x}$ NC solution was measured with inductively coupled plasma optical emission spectrometry (ICP-OES). Thus, the $CsPbBr_xCl_{3-x}$ NC concentration can be obtained as n_2/ n_1, which can be used to calculate the extinction coefficient according to Lambert-Beer law.

Estimation of FRET rate

The EnT rate from photoexcited NCs to TCAs was estimated using Förster Resonant Energy Transfer (FRET) model based on dipole approximation. According to this model, the Förster radius (R), the distance between donor and acceptor at which the emission of the donor is

quenched by 50%, is given by the following equation:

$$R = 0.0211[\kappa^2 \Phi_D J(\lambda)/n^4]^{1/6} \quad \text{(S1)},$$

where κ^2 is dipole orientation factor which 2/3 for randomly oriented dipoles, Φ_D is emission quantum yield of the donor which is 25% for our NCs , $J(\lambda)$ is the overlap integral between the donor emission and the acceptor absorption, and n is the refractive index of dielectric medium. The overlap integral can be calculated using:

$$J(\lambda) = \frac{\int d\lambda I_D(\lambda)\varepsilon_A(\lambda)(\lambda)^4}{\int d\lambda I_D(\lambda)} \quad \text{(S2)},$$

where $I_D(\lambda)$ and $\varepsilon_A(\lambda)$ are the donor emission spectrum and acceptor molar absorptivity, respectively. Based on the absorption and emission spectra of NCs and TCAs, $J(\lambda)$ is calculated to be 3.13×10^{14} $M^{-1}cm^{-1}nm^4$. As such, the Förster radius R is calculated to be 3.28 nm. With this, the EnT time constant can be calculated as:

$$\tau_{EnT} = \tau_0 (\frac{r}{R})^6 \quad \text{(S3)},$$

where τ_0 is the donor excited state lifetime (~5.03 ns for NCs) and r is the donor-acceptor distance (~5 nm for NC-TCA complexes). Therefore, we can estimate that the EnT time constant from one NC to one TCA molecule is ~63 ns. Considering that there are ~137 TCA acceptors for one NC, the realistic EnT time for our NC-TCA complexes is ~0.46 ns.

Estimation of average exciton number at each excitation density

This estimation (Figure 3a inset) was done by fitting the scaled XB signal at a delay time of 200 ps measured as a function of the excitation fluence to a Poisson statistics model. According to this model, the photon absorption events when using above band-gap excitation

follows a Poisson distribution,[6] and hence, the XB signal at a delay time of 200 ps can be expressed as: $I \propto 1 - P(0) = 1 - e^{-<N>}$, where $<N>$ is the average number of photons absorbed per QD or the average exciton number per QD prior to Auger recombination. $<N>$ is proportional to the pump laser fluence: $<N> = Cj$. Therefore, the XB signal at 200 ps can be fitted with: $I \propto 1 - e^{-Cj}$, with C as the only fitting parameter. Using the fitted C value, the average exciton number $<N>$ at each pump fluence can be calculated.

Estimation of exciton dissociation number

We estimate the upper limit for the number of dissociated excitons per NC by making two assumptions. First, the HT time maintains constant, *i.e.*, it is not affected by previously dissociated excitons; second, Auger recombination follows a sequential, staircase-like behavior, *i.e.*, N-exciton state decays to (N-1)-exciton state and is generated by the decay of (N+1)-exciton state, and the lifetime of N-exciton state is related to the biexciton state *via*: $\tau_{NX} = 2\tau_{XX}/N(N\text{-}1)$.[7] This corresponds to the situation of simultaneous multi-hole transfer to multiple acceptors. The situation of sequential multi-hole transfer would involve the generation of charged multiexciton states and charge transfer from charged excitons, which is too complicated to be simply accounted for. Based on above assumptions, the number of dissociated excitons (n) from an N-exciton state can be calculated as:

$$n = \sum_{i=2}^{N} \frac{\tau_{NX}}{\tau_{NX} + \tau_{HT}} N + 1 = \sum_{i=2}^{N} \frac{2\tau_{XX} / [i(i-1)]}{2\tau_{XX} / [i(i-1)] + \tau_{HT}} N + 1 \qquad \text{(S4)},$$

where the factor 1 accounts for the unity-yield dissociation of single-exciton state. Considering the Poisson distribution of exciton number in an ensemble of photoexcited NCs, the average number of dissociated excitons ($<n>$) from an ensemble with average exciton

number of <*N*> is:

$$\langle n\rangle = P(1)+\sum_{N=2}^{\infty}P(N)\left\{\sum_{i=2}^{N}\frac{2\tau_{XX}/\left[i(i-1)\right]}{2\tau_{XX}/\left[i(i-1)\right]+\tau_{HT}}N+1\right\} \qquad \text{(S5)},$$

where *P*(*N*) is the possibility of finding an *N*-exciton state when the average exciton number is <*N*>. Using eq. S5, the average number of dissociated excitons <*n*> as a function of average exciton number <*N*> can be calculated, which is plotted is Figure S7. <*n*> saturates at a value of ~6.5.

In reality, however, <*n*> should be lower as the sequential multi-hole transfer pathway mentioned above should also exist. In this case, HT rate should be affected by previous dissociated excitons as previous dissociation results in charging energy for HT and also reduces the number of available acceptors. In addition, HT has to compete with Auger recombination of charged multiexcitons in this case. As a result, our experimentally observed <*n*> is smaller (5.6). However, the small difference between the estimated upper limit and measured value suggests that simultaneous multi-hole transfer is likely the major channel for multiexciton dissociation.

Reference.

(1) Wu, K.; Liang, G.; Shang, Q.; Ren, Y.; Kong, D.; Lian, T. Ultrafast Interfacial Electron and Hole Transfer from CsPbBr3 Perovskite Quantum Dots. *J. Am. Chem. Soc.* **2015,** *137*, 12792-12795.

(2) Wang, J.; Ding, T.; Leng, J.; Jin, S.; Wu, K. “Intact” Carrier Doping by Pump–Pump–Probe Spectroscopy in Combination with Interfacial Charge Transfer: A Case Study of CsPbBr3 Nanocrystals. *J. Phys. Chem. Lett.* **2018,** *9*, 3372-3377.

(3) Protesescu, L.; Yakunin, S.; Bodnarchuk, M. I.; Krieg, F.; Caputo, R.; Hendon, C. H.; Yang, R. X.; Walsh, A.; Kovalenko, M. V. Nanocrystals of Cesium Lead Halide Perovskites ($CsPbX_3$, X = Cl, Br, and I): Novel Optoelectronic Materials Showing Bright Emission with Wide Color Gamut. *Nano Lett.* **2015**, 15, 3692-3696.

(4) Müller A. M.; Avlasevich Y. S.; Schoeller W. W.; Müllen, K.; Bardeen, C. J. Exciton Fission and Fusion in Bis(tetracene) Molecules with Different Covalent Linker Structures. *J. Am. Chem. Soc.* **2007**, 129, 14240-14250.

(5) Okamoto, T.; Suzuki, T.; Tanaka, H.; Hashizume, D.; Matsuo, Y. Tetracene Dicarboxylic Imide and Its Disulfide: Synthesis of Ambipolar Organic Semiconductors for Organic Photovoltaic Cells. *Chem.- Asia. Jour.*, **2012**, 7, 105-111

(6) Klimov, V. I.; Mikhailovsky, A. A.; McBranch, D. W.; Leatherdale, C. A.; Bawendi, M. G. Quantization of multiparticle Auger rates in semiconductor quantum dots. *Science* **2000,** *287*, 1011-1013.

(7) Klimov, V. I. Multicarrier Interactions in Semiconductor Nanocrystals in Relation to the Phenomena of Auger Recombination and Carrier Multiplication. *Ann. Rev. Cond. Matt. Phy.* **2014,** *5*, 285-316.
